# Simultaneous acute limb ischemia related to acute Leriche syndrome and pulmonary embolism without existing patent foramen ovale: a case report

**DOI:** 10.1186/s12872-021-02272-3

**Published:** 2021-09-26

**Authors:** Ying-Sheng Li, Ying-Ching Li

**Affiliations:** Division of Thoracic and Cardiovascular Surgery, Department of Surgery, Chang Gung Memorial Hospital at Linkou, Chang Gung University, Taoyuan, Taiwan

**Keywords:** Acute Leriche syndrome, Pulmonary embolism, Aortoiliac occlusion, Intracardiac right-to-left shunt, Patent foramen ovale

## Abstract

**Background:**

Aortoiliac occlusion disease, also called Leriche syndrome, is characterized by atherothrombotic obliteration of the aortic bifurcation and bilateral common iliac arteries; typically, it has a chronic presentation. Pulmonary embolism is more related to venous thromboembolism rather than arterial thromboembolic events. Therefore, cases of simultaneous acute Leriche syndrome and pulmonary embolism are rare. Existing intracardiac right-to-left shunt were detected in most previous cases. Herein, we present the first likely documented case wherein acute Leriche syndrome and pulmonary embolism occurred simultaneously without a patent foramen ovale.

**Case presentation:**

A 58-year-old man with hyperlipidemia and coronary artery disease presented with a 4-h history of bilateral lower limb numbness. He was a heavy smoker with a history of stroke. Computed tomography angiography revealed pulmonary embolism and aortoiliac artery occlusion. Although a massive thrombus straddled the bilateral pulmonary arteries, orthopnea was his only presentation, without right ventricle failure. Cyanosis of the affected limbs was noted, and muscle strength in both limbs had regressed to grade 1. Owing to acute limb ischemia, he underwent an emergency operation to salvage the limbs. On postoperative day 5, the general condition of both the legs improved; the muscle strength improved to grade 4. He was then transferred to the general ward and enoxaparin was continued. Computed tomography angiography was repeated to evaluate the pulmonary embolism on postoperative day 8; the thrombus remained lodged in the bilateral main pulmonary arteries. Owing to persistent orthopnea and chest tightness with intermittent tachycardia, he underwent a staged operation for the pulmonary embolism on postoperative day 13. During the surgery, intraoperative transesophageal echocardiography showed no patent foramen ovale or an existing right-to-left shunt. Postoperatively, he was closely monitored in the intensive care unit for 3 days and then transferred to the general ward for 10 days. A final computed tomography angiography performed on postoperative day 18 revealed thrombus resolution. He was then discharged on postoperative day 30 without any in-hospital complications.

**Conclusion:**

We present a case that might be the first documented report of acute Leriche syndrome co-occurring with pulmonary embolism without an existing patent foramen ovale.

## Background

Acute limb ischemia (ALI) and pulmonary embolism (PE) are critical illnesses that may result in severe morbidity and mortality for patients [1]. Aortoiliac occlusion disease, also called Leriche syndrome, is characterized by atherothrombotic obliteration of the aortic bifurcation and bilateral common iliac arteries. The disease is typically chronic but rarely causes acute symptoms unlike ALI [2]. PE, as a type of venous thromboembolism, is less likely to be associated with arterial thromboembolic events, except paradoxical embolism [3, 4]. Herein, we present the case of a patient with concomitant PE and ALI associated with acute Leriche syndrome without an existing intracardiac right-to-left shunt. To the best of our knowledge, this is the first documented case of the co-occurrence of an aortoiliac occlusion and PE without the presence of a patent foramen ovale (PFO).

## Case presentation

**Fig. 1:**
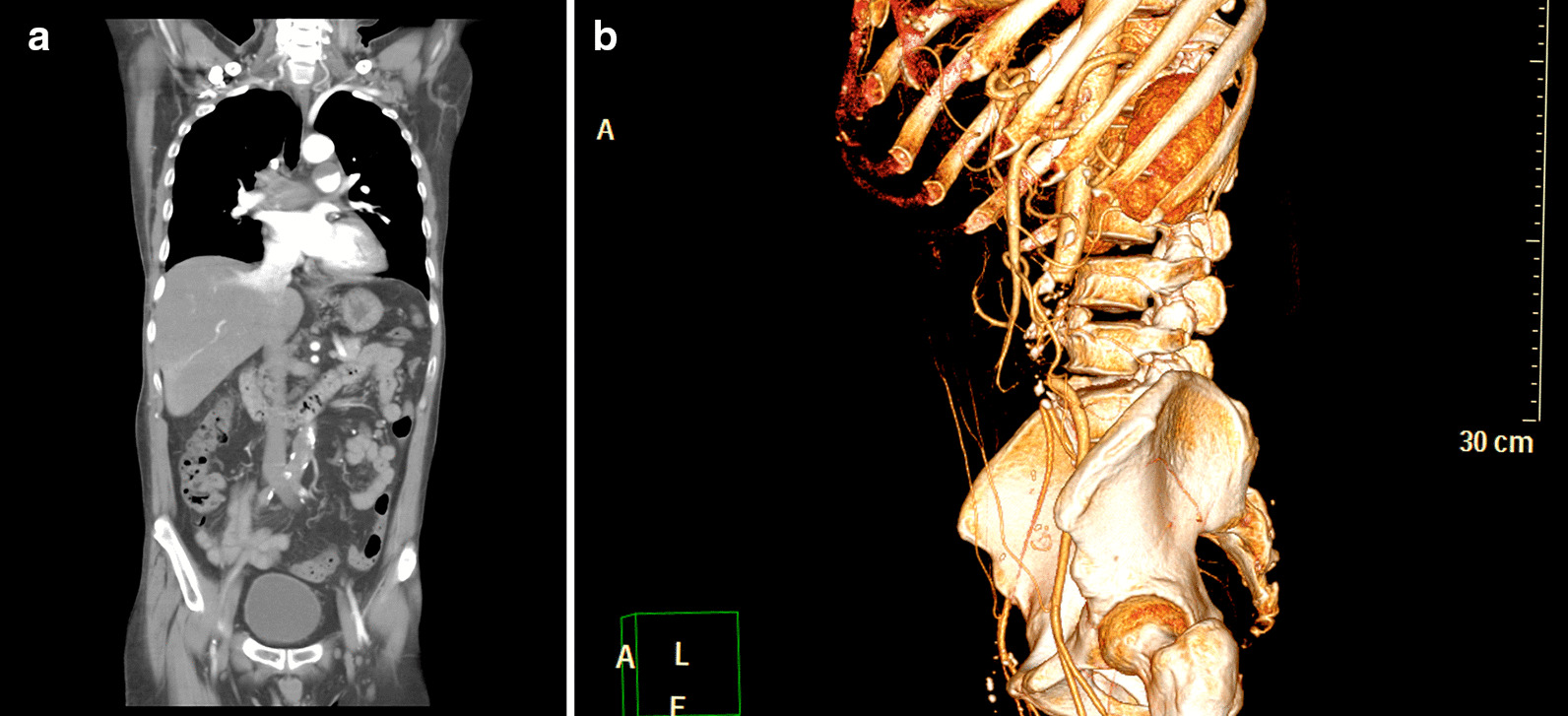
Computed tomography angiography shows **a** a massive thrombus straddling both the pulmonary arteries, with aortoiliac occlusion disease. **b** However, there is no obvious establishment of collateral vessels around the occluded vessels from the infrarenal abdominal aorta to the bilateral common iliac arteries

**Fig. 2:**
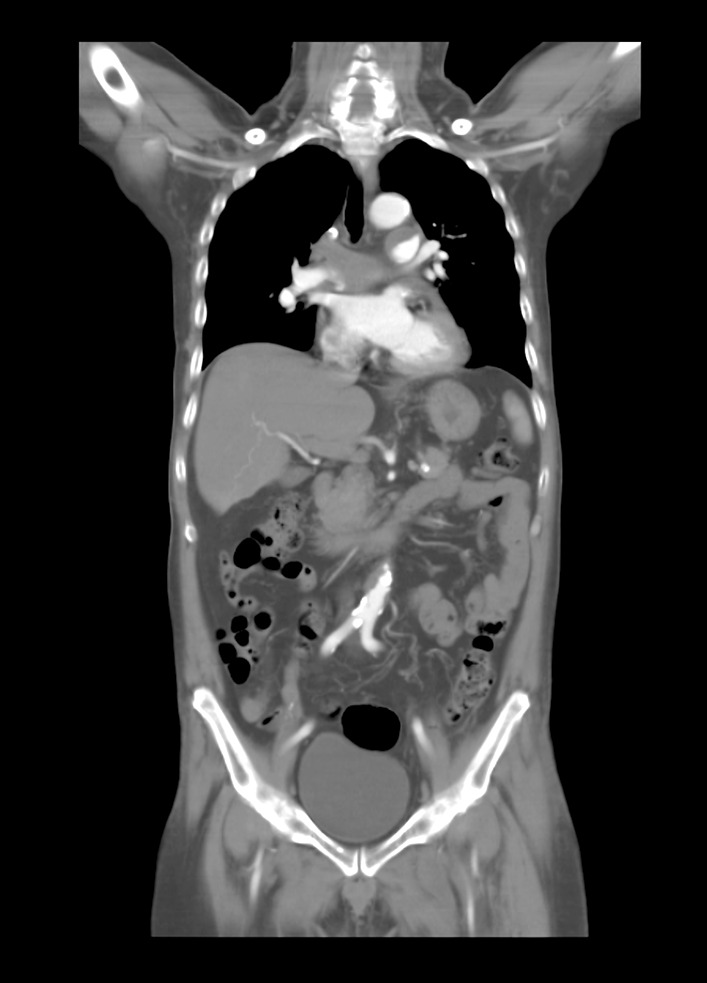
Computed tomography angiography was performed again for further evaluation on postoperative day 8; the results reveal that the thrombus was still lodged in the bilateral main pulmonary arteries, although revascularization of the aortoiliac arteries is seen

**Fig. 3:**
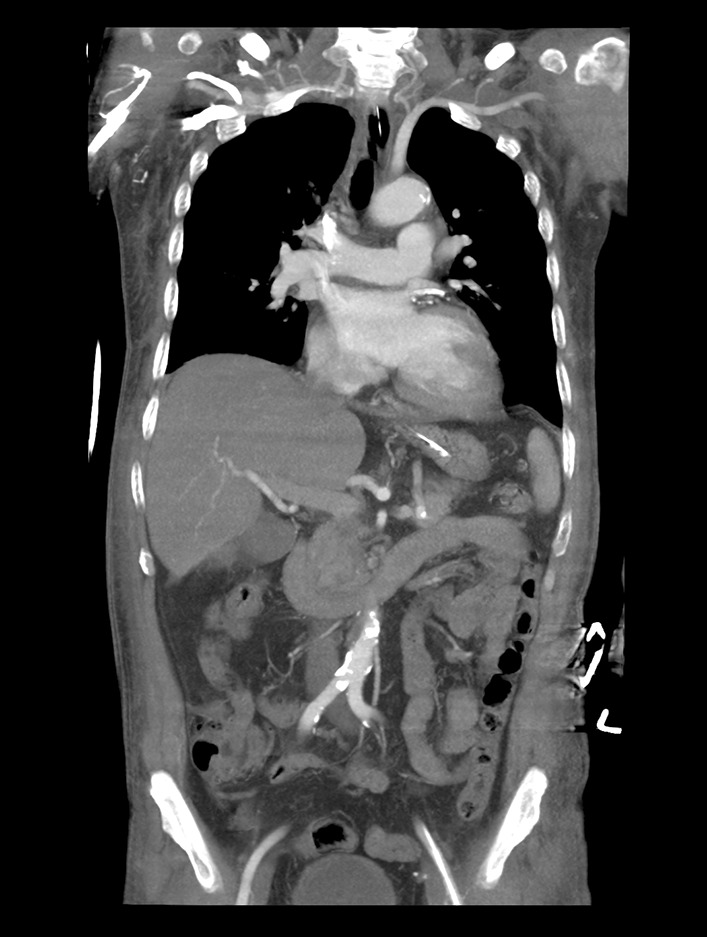
The final computed tomography angiography reveals no residual thrombus burden in both the pulmonary arteries

A 58-year-old male patient with hyperlipidemia, and maintained on dual antiplatelet therapy (DAPT) due to coronary artery disease status and implantation of two stents, presented with a 4-h history of bilateral lower limb numbness. He was also a heavy smoker and had a history of stroke. He was initially sent to the local medical department where a computed tomography angiography (CTA) revealed pulmonary embolism with a massive thrombus straddling both the pulmonary arteries and aortoiliac artery occlusion with poor collateral recanalization to the lower limb arteries (Fig. 1). He was immediately transferred to our center with symptoms of mild orthopnea and resting pain. In addition, cyanosis of the affected limbs was noted, and muscle strength in both the limbs regressed to grade 1. Since enoxaparin was given before transfer and echocardiography did not reveal right ventricle failure, he underwent an emergency thrombectomy for the aortoiliac artery occlusion. After the first operation to salvage the limbs, he was admitted to the intensive care unit for further treatment. On postoperative day 5, the general condition of both the legs improved, with the muscle strength improving to grade 4. He was then transferred to the general ward, and DAPT and enoxaparin were continued to be administered. Owing to the persistence of orthopnea and complaint of chest tightness with intermittent tachycardia, a CTA was performed again to further evaluate the pulmonary embolism on postoperative day 8 (Fig. 2). The CTA results revealed that the thrombus was still lodged in the bilateral main pulmonary arteries. Fearing the possibility of a myocardial infarction, cardiac catheterization was performed, which did not reveal any intrastent restenosis or specific lesions in the coronary arteries. Owing to the failure of medical treatment, a staged pulmonary embolectomy with cardiac arrest using heart–lung-machine support was performed on postoperative day 13. During the surgery, intraoperative transesophageal echocardiography did not reveal a PFO or an existing right-to-left shunt. Postoperatively, he was closely monitored in the intensive care unit for 3 days and then transferred to the general ward for 10 days. A follow-up CTA was performed on postoperative day 18 (Fig. 3), which revealed resolution of the thrombus in the pulmonary arteries. He was discharged on postoperative day 30 under DAPT and rivaroxaban prescription, without any in-hospital complications.

## Discussion and conclusion

This is an unusual case that seldom presents in our clinical practice. Aortoiliac occlusive disorder, which is often related to an atherosclerotic disease, usually presents as chronic pain in the buttocks and pain during walking. The classical triad of clinical symptoms is intermittent claudication, impotence in males, and weak or absent femoral pulses [5]. In this case, the patient complained of sudden onset of bilateral leg pain and paralysis while sleeping without the triad of symptoms. Because of the chronicity of the disease, the collateral vessels, or the so-called choke vessels, were always present along with the slowly-developing occlusions. However, there was no obvious establishment of collateral vessels around the occluded arteries in our patient. In addition, unlike previous reports, the aortoiliac bifurcation was not severely calcified [5]. Therefore, instead of a chronic clinical course, we considered a case of acute Leriche syndrome.

PE may be a life-threatening condition if there is an acute attack, and emergency surgical intervention might be needed if the patient’s vital signs remain unstable [1]. In our case, echocardiography did not reveal right-sided ventricle failure, although CTA revealed a massive thrombus in the bilateral main pulmonary arteries. Orthopnea was the only presentation in our patient, and inotropic drugs were not administered. Our treatment strategy was to salvage his limbs and administer anticoagulation therapy for his PE.

In most cases, ALI and PE do not co-occur because of the difference in the arterial and venous systems, unless a “bridge” is present between the two systems. A paradoxical embolism refers to the embolic entry of a venous thrombus into the systemic circulation through a right-to-left shunt, such as a PFO [3]. The diagnosis includes the presence of (1) a systemic arterial embolus that does not arise from the left side of the heart, (2) venous thrombosis and/or pulmonary embolus, and (3) an intracardiac communication such as a right-to-left shunt [4, 6]. With a prevalence around 25–30% in the general population, the incidence of PFO remains underestimated. However, according to Gouëffic et al. [3], only few patients have been reported to experience an atherothrombotic occlusion in the aortic bifurcation due to a paradoxical embolism.

Even without a PFO, an aortoiliac artery occlusion with concomitant PE may still occur. In our presented case, acute Leriche syndrome and PE were documented by the CTA initially. Under the suspicion of a PFO, a series of studies were performed, including transthoracic echocardiography, cardiac catheterization, and intraoperative transesophageal echocardiography, during the second operation. However, there was no evidence or sign of a right-to left shunt. Although there is no specific reason or explanation for the co-occurrence of an aortoiliac artery occlusion with PE in our patient, he still underwent two staged operations for ALI and PE. The postoperative CTA showed resolution of most of the thrombus in the main pulmonary arteries and bilateral aortoiliac arteries.

In conclusion, Leriche syndrome is typically a chronic disease caused by aortoiliac artery occlusion but can sometimes occur within a short span of time. The most common cause of acute Leriche syndrome is a combination of PE and a PFO. However, the reasons for sudden onset of aortoiliac occlusion with concomitant PE in some cases may remain unknown. The patient presented in this case may be the first documented case of acute Leriche syndrome co-occurring with PE without an existing PFO.

## Data Availability

Data sharing is not applicable to this article as no datasets were generated or analyzed during the current study.

## Abbreviations

ALI: Acute limb ischemia
PE: Pulmonary embolism
PFO: Patent foramen ovale
DAPT: Dual antiplatelet therapy
CTA: Computed tomography angiography
